# Case Report: Scalpel Sign and Dorsal Arachnoid Cyst—The Importance of an Accurate Diagnosis

**DOI:** 10.3390/reports8040198

**Published:** 2025-10-05

**Authors:** Matteo Bonetti, Michele Frigerio, Mario Muto, Federico Maffezzoni, Serena Miglio

**Affiliations:** 1 Department of Neuroradiology, Istituto Clinico Città di Brescia, 25128 Brescia, Italy; michele.frigerio@gmail.com; 2 Department of Neuroradiology, Ospedale Cardarelli, Via Antonio Cardarelli 9, 80131 Napoli, Italy; mario.muto@aocardarelli.it; 3 Oberdan Specialist Outpatient Clinic, Via Guglielmo Oberdan 126, 25128 Brescia, Italy; dott.federicomaffezzoni@gmail.com (F.M.); serenamiglio.dottssa@gmail.com (S.M.)

**Keywords:** scalpel sign, cord compression, ventral cord herniation, arachnoid web, arachnoid cyst

## Abstract

**Background and Clinical Significance**: Thoracic dorsal arachnoid web (DAW) is a rare intradural extramedullary condition characterized by a thin band of arachnoid tissue compressing the dorsal spinal cord. A hallmark imaging feature is the “scalpel sign”, which refers to anterior displacement of the thoracic spinal cord with dorsal cerebrospinal fluid (CSF) accumulation, producing a sagittal profile resembling a surgical scalpel. Although highly specific for DAW, this sign may also appear in other intradural conditions such as idiopathic ventral spinal cord herniation and arachnoid cysts. The clinical presentation is typically progressive and nonspecific, including lower limb weakness, sensory changes, gait disturbances, and, less frequently, sphincter dysfunction. Diagnosis is often delayed due to the subtle nature of the lesion and limited resolution of conventional Magnetic Resonance Imaging (MRI). High-resolution Three-Dimensional Constructive Interference in Steady State (3D-CISS) sequences improve diagnostic accuracy by highlighting indirect signs such as spinal cord deformation and dorsal CSF flow obstruction. **Case Presentation**: We report the case of a 57-year-old woman presenting with chronic cervico-dorsalgia, bilateral lower limb weakness, paresthesia, and progressive gait instability. Neurological examination revealed spastic paraparesis and hyperreflexia. Conventional MRI was inconclusive. However, sagittal T2-weighted and 3D-CISS sequences demonstrated the scalpel sign at the T4–T5 level, with anterior cord displacement and dorsal subarachnoid space enlargement. Surgical exploration confirmed the presence of a dorsal arachnoid web, which was resected. Postoperative follow-up showed clear improvement in motor function and gait. **Conclusions**: DAW should be considered in cases of unexplained thoracic myelopathy or cervico-dorsalgia with neurological signs. Early recognition of the scalpel sign using advanced MRI sequences is critical for timely diagnosis and surgical planning, which may lead to significant clinical improvement.

## 1. Introduction and Clinical Significance

The “scalpel sign” was first described by Reardon et al. [1] as a pathognomonic imaging finding in various intradural extramedullary spinal pathologies, in the absence of overt lesions such as tumors, hemorrhages, or infections [1,2]. The term refers to the anterior displacement of the thoracic spinal cord, resulting in a prominent dorsal collection of cerebrospinal fluid (CSF), which appears on sagittal imaging as a silhouette reminiscent of a surgical scalpel blade. This sign has been associated with several conditions, including thoracic dorsal arachnoid web (DAW), idiopathic ventral spinal cord herniation, and intradural spinal arachnoid cysts [1,2,3,4,5,6,7,8].

Dorsal arachnoid web (DAW) is an intradural extramedullary band of arachnoid tissue along the dorsal surface of the spinal cord, typically in the upper thoracic region, causing focal compression often associated with the scalpel sign [1,2,3,4,5].

The clinical presentation usually includes progressive bilateral lower limb weakness, sensory disturbances, and gait impairment, sometimes accompanied by dorsal neuropathic pain or, less commonly, sphincter dysfunction. Neurological findings may involve hyperreflexia, spastic paraparesis, clonus, and gait instability [1,2,6].

Although only 63 cases had been documented up to July 2021 [5,9,10,11,12,13,14,15,16], a recent systematic review has expanded this number to 197 surgically confirmed cases, reflecting the growing awareness and clinical interest surrounding this condition [17]. Yet, despite this increasing recognition, the overall number of reported cases remains limited across the literature, highlighting the need for further documentation and deeper radiological characterization [17].

Importantly, DAW and the scalpel sign are closely related but not synonymous. DAW represents the underlying anatomical lesion, whereas the scalpel sign is an imaging marker defined by anterior displacement of the spinal cord with dorsal cerebrospinal fluid accumulation on sagittal MRI. Although the scalpel sign is highly suggestive of DAW and frequently reported in surgically confirmed cases, it has also been described in other intradural extramedullary conditions such as arachnoid cysts or ventral spinal cord herniation. Conversely, DAW may rarely occur without a detectable scalpel sign, particularly when imaging resolution is suboptimal (Table 1).

Although patient history may occasionally include prior surgical procedures, trauma, or central nervous system infections, the etiology of scalpel sign often remains unclear, making it difficult to establish a definitive causal relationship.

Gender distribution is still debated: some case series report a female predominance with a 2:1 ratio, mainly affecting individuals between the fourth and seventh decades of life [1,2], whereas a more recent review of 41 cases reported a mean age of onset of 52 years and a higher incidence among males, with a male-to-female ratio of 2.6:1 [5].

The diagnostic workup is often complex, primarily due to the thin and poorly distinguishable nature of the arachnoid membrane. Magnetic Resonance Imaging (MRI) remains the diagnostic modality of choice, although the limited resolution of conventional sequences can hinder direct visualization of the lesion relative to surrounding structures. Yamaguchi et al. [7] demonstrated that MRI often suggests arachnoid abnormalities only indirectly, through deformation of the spinal cord and disruption of CSF flow [27].

However, sagittal high-resolution T2-weighted sequences may highlight two key features:

- A transverse extramedullary dorsal arachnoid band;
- A focal indentation of the spinal cord.

Given the limited sensitivity of conventional MRI in detecting the arachnoid membrane, advanced imaging techniques have been introduced to enhance diagnostic accuracy and anatomical definition. The Three-Dimensional Constructive Interference in Steady State (3D-CISS) sequence has proven more effective in visualizing membranes, particularly in cases where CT myelography was only suggestive [27].

The concurrent presence of these imaging features defines the scalpel sign, a finding regarded as highly specific for DAW. On sagittal MRI, the focal dorsal indentation and anterior displacement of the spinal cord create a silhouette resembling a posteriorly directed surgical blade, hence the name. [3,7,27,28,29,30,31,32,33,34,35,36,37].

The pathogenesis of DAW remains incompletely understood. Leading hypotheses include:

- Forced CSF flow through a congenital dural defect;
- Post-traumatic, post-infectious, or postoperative processes [1,9,30];
- A residual or variant form of a collapsed arachnoid cyst [1,30].

In the absence of identifiable predisposing factors, an idiopathic or congenital origin is also considered [1,10].

MRI remains the reference imaging modality, whereas Computed Tomography (CT) myelography is now rarely used [1,30]. DAW may be misdiagnosed as an arachnoid cyst, typically a well-defined lesion with slow filling on dynamic sequences and broader cord distortion, usually lacking the classic scalpel sign [5,30]. Idiopathic ventral spinal cord herniation is another key differential diagnosis, characterized by ventral cord displacement, interruption of the anterior subarachnoid space, and a dorsally concave (“C-shaped”) cord surface; surgical repair of the anterior dural defect is the treatment of choice [1,3].

This case report aims to contribute to the limited literature on the scalpel sign. Despite its diagnostic value, current evidence remains scarce, particularly regarding its detection in patients with inconclusive conventional MRI findings and its potential relationship with arachnoid cysts. By presenting this case, we highlight the role of advanced MRI techniques, such as 3D-CISS, in improving diagnostic accuracy and addressing existing gaps in the characterization and clinical understanding of this rare entity.

## 2. Case Presentation

A 57-year-old woman M.A.M., was referred for evaluation of progressive cervico-dorsal pain, bilateral lower limb weakness, paresthesia, and unsteady gait. Neurological examination revealed signs of upper motor neuron involvement, including spastic paraparesis and hyperreflexia. There was no history of trauma, neurosurgical procedures, or central nervous system infections. The patient’s general medical history was unremarkable.

**Figure 1:**
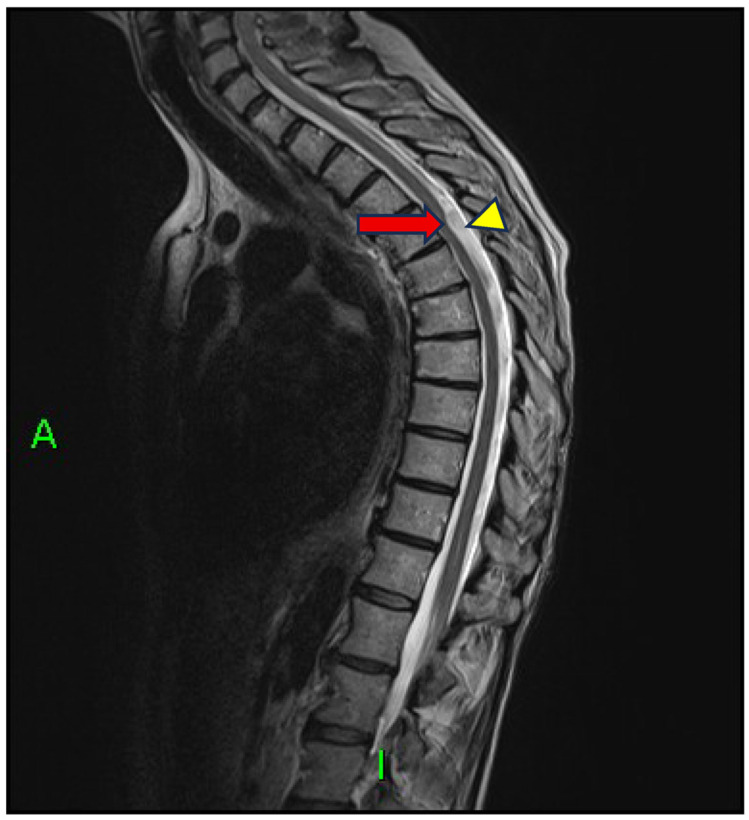
Sagittal T2-weighted MRI (20 × 3 mm; Gap: 10%; TR: 4930 ms; TE: 93 ms; FOV: 320 mm; Matrix: 320 Pd HF) showing a characteristic focal dorsal indentation and ventral displacement of the dorsal spinal cord at the T4–T5 level (red arrow), accompanied by enlargement of the dorsal subarachnoid space (yellow arrow tip). This configuration resembles the shape of a surgical blade, commonly referred to as the scalpel sign.

Initial spinal MRI with conventional sequences failed to reveal any compressive lesion or space-occupying pathology. However, sagittal T2-weighted images demonstrated a focal dorsal indentation of the spinal cord at the T4–T5 level, associated with anterior displacement of the thoracic cord and an enlarged dorsal cerebrospinal fluid (CSF) space—an imaging pattern consistent with the scalpel sign (Figure 1).

**Figure 2:**
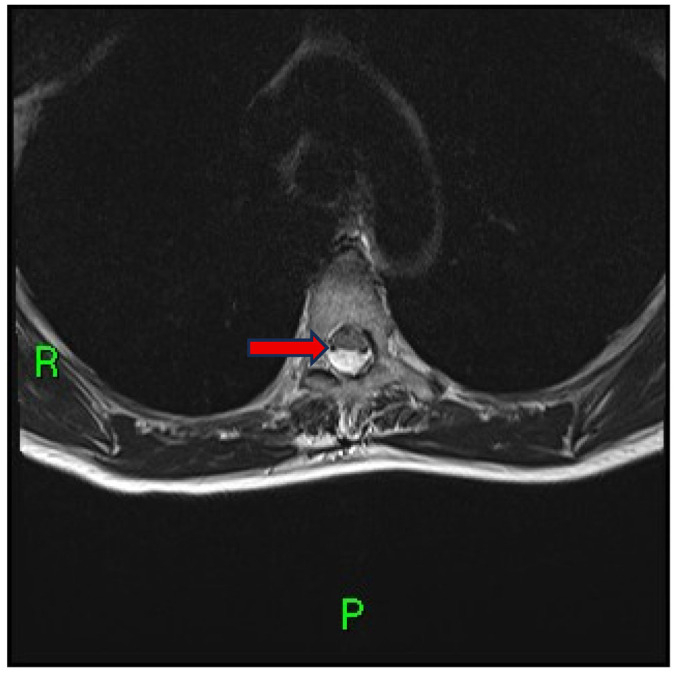
Axial T2-weighted MRI (35 × 4 mm; Gap: 10%; TR: 7480 ms; TE: 102 ms; FOV: 240 mm; Matrix: 320 Pd AP) above the dorsal indentation at the T5 level demonstrates an enlarged posterior subarachnoid space and ventral displacement of the dorsal spinal cord (arrow).

Subsequent axial imaging obtained just above the level of indentation confirmed ventral displacement of the dorsal spinal cord and enlargement of the posterior subarachnoid space (Figure 2).

**Figure 3:**
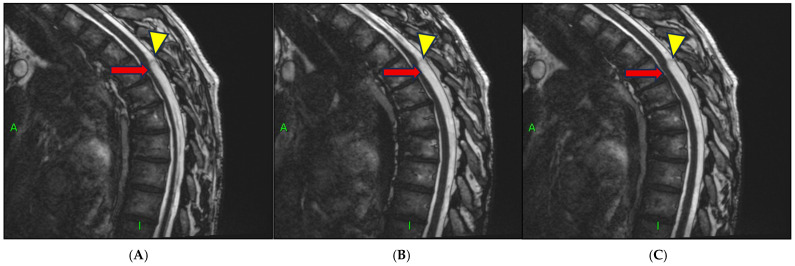
(**A**–**C**) Sagittal 3D-CISS MRI (52 × 0.9 mm; Gap: 0%; TR: 5.19 ms; TE: 2.29 ms; FOV: 250 mm; Matrix: 448 Pd HF) demonstrates focal distortion of the thoracic spinal cord with anterior displacement (red arrows), consistent with the scalpel sign. The dome-shaped contour of the arachnoid cyst is also well visualized (yellow arrow tips). (**A**–**C**) represent three consecutive sagittal 3D-CISS sequences obtained in series, sequentially depicting the progressive visualization of the characteristic scalpel sign on MRI.

High-resolution 3D-CISS MRI (Figure 3A–C) sequences additionally revealed a well-demarcated, dome-shaped lesion suggestive of an arachnoid cyst adjacent to the site of cord compression (Figure 3A–C). This finding supported the hypothesis of a coexisting arachnoid cyst, either contributing to the mass effect or representing a collapsed structure related to the dorsal arachnoid web (DAW).

## 3. Discussion

The scalpel sign represents a highly suggestive neuroradiological finding, detectable in a limited number of intradural extramedullary conditions located in the thoracic spine, including DAW and intradural arachnoid cysts [1,2,3]. Its identification assumes critical diagnostic value, particularly in cases of non-specific thoracic myelopathic symptoms, characterized by progressive motor impairment and sensory disturbances in the absence of overt lesions [4,20]. The high specificity of this sign necessitates thorough neuroradiological assessment, as failure to recognize it may lead to significant delays in the diagnostic and therapeutic process [5,10].

MRI with 3D-CISS sequences significantly improves the detection of fine arachnoid structures, often indistinct on conventional imaging, by suppressing CSF signal and providing high spatial resolution [28,37]. This enables clear visualization of the dorsal spinal cord morphology and the membranous bands responsible for the characteristic scalpel sign, defined by focal indentation and anterior cord displacement [1.4]. Moreover, 3D-CISS allows dynamic assessment of CSF flow, where unidirectional obstruction in DAW or collapsed cysts represents a highly suggestive diagnostic feature [7,27].

High-resolution 3D-CISS imaging provides clearer depiction of thin arachnoid membranes that are often indistinct on conventional MRI [17,20,28]. This improved anatomical definition enhances diagnostic accuracy by refining the differential among intradural extramedullary lesions and by defining the exact level and extent of pathology [3,5]. It also informs surgical decision-making: for dorsal arachnoid webs, localized resection or limited posterior decompression typically yields favorable outcomes [12,18,20] whereas dorsal arachnoid cysts may require fenestration or complete excision—and, in selected recurrences, shunting— to prevent relapse [38,39,40]. In scenarios with pronounced ventral displacement consistent with idiopathic spinal cord herniation, posterior decompression alone is often inadequate and repair of the anterior dural defect may be indicated [3]. Finally, detailed postoperative imaging, including the use of CISS where available, can help detect residual membranes or adhesions and support long-term follow-up [14,38,41].

Differential diagnosis requires accurate morpho-functional imaging. Intradural arachnoid cysts usually show well-defined margins, slow contrast filling, and symmetric spinal cord deformation, whereas DAW appears as a thin, isolated membrane often difficult to visualize without high-resolution sequences [5,9]. Idiopathic ventral spinal cord herniation, instead, presents with anterior displacement, posterior cord contour distortion, and ventral subarachnoid space interruption [3,42]. These imaging distinctions are clinically relevant, as they guide different surgical strategies [12,18].

Delayed or inaccurate diagnosis may result in inappropriate therapeutic decisions and progressive spinal cord injury. In symptomatic cases, surgical intervention remains the only effective strategy to achieve lasting decompression and prevent chronic myelopathy [12,18,19].

Alterations in CSF flow secondary to arachnoid membrane thickening may contribute to the development of syringomyelia, most frequently cranial to the level of the web, although caudal cases have also been described [1,43]. Delayed diagnosis has been associated with progressive spinal cord damage, worsening sensory-motor deficits, and risk of permanent impairment, whereas early recognition with targeted decompression may promote syrinx regression or stabilization and improved neurological outcomes [44,45,46]. Moreover, accurate differentiation from ventral spinal cord herniation and arachnoid cysts is crucial, as inadequate surgical strategies may fail to restore CSF flow and perpetuate the syrinx [2,3,42]. The systematic use of high-resolution sequences (3D-CISS) and CSF flow assessment facilitates the identification of thin membranes and obstruction patterns, enabling timely treatment [1,7,29,43,44,45,46].

The scalpel sign is a clinically relevant neuroradiological marker whose accurate detection relies on high-resolution MRI, CSF flow analysis, and specialized expertise to ensure precise diagnosis and optimal treatment planning [1,17,28]

This case adds to the limited but growing body of literature highlighting the diagnostic value of the scalpel sign in thoracic spinal pathologies [1,4,20]. and reinforces the clinical importance of incorporating high-resolution and dynamic MRI protocols in patients with unexplained thoracic myelopathy [28,38]. The identification of a DAW, particularly in the absence of clear compressive lesions on conventional imaging, requires a high index of suspicion and specific neuroradiological expertise [3,5]. The concurrent visualization of a dome-shaped arachnoid cyst in our patient further underscores the possibility of a pathogenetic continuum between DAW and arachnoid cysts [39,40]. suggesting that these may not represent entirely distinct entities but rather different expressions of the same underlying arachnoid anomaly [9].

From a clinical standpoint, early recognition of the scalpel sign may significantly reduce diagnostic delays and facilitate timely surgical intervention, thereby improving long-term neurological outcomes [9,18,22]. Our findings also highlight the utility of 3D-CISS sequences not only in visualizing subtle arachnoid membranes but also in assessing CSF flow dynamics [7,27] which may offer additional diagnostic and prognostic insights [30].

Future research should aim to systematically investigate the etiological and morphological spectrum of DAW and its potential overlap with arachnoid cysts [17,19]. Multicenter prospective studies with larger sample sizes and standardized imaging protocols are needed to validate current diagnostic criteria, refine differential diagnoses, and optimize surgical planning [20]. Moreover, longitudinal studies evaluating postoperative outcomes could help establish prognostic indicators and contribute to the development of evidence-based guidelines for the management of intradural extramedullary thoracic pathologies [14,22]. This case contributes to consolidating and expanding the knowledge base of a condition that remains underdiagnosed poorly under-represented in the existing literature. In a field where available evidence is still limited and fragmented, each report holds cumulative value, helping to refine diagnostic criteria, guide therapeutic strategies, and encourage the development of larger multicenter prospective studies. For this reason, a greater awareness of the relationships between often coexisting anatomical manifestations is needed. Given the frequent coexistence between DAW and the scalpel sign (Table 1), further investigation through comparative analyses across anatomically related entities (e.g., DAW, arachnoid cysts, ventral cord herniation) would be warranted. By broadening the current evidence base, the present report may serve as a preliminary step toward a more integrated understanding of these interrelated pathologies in future multicenter research.

## 4. Conclusions

Early identification of the scalpel sign using high-resolution MRI sequences such as 3D-CISS provides decisive evidence when conventional imaging is inconclusive. Incorporating this finding into a comprehensive morpho-functional assessment enhances diagnostic accuracy and supports timely, well-targeted clinical and surgical decision-making for intradural extramedullary lesions.

## Figures and Tables

**Table 1:** Case reports, case series and systematic reviews addressing DAW and the scalpel sign, identified primarily through a PubMed literature search and supplemented by pertinent publications. The table summarizes the year, first author, journal, number of cases, lesion type, and principal findings, providing an updated synthesis of clinically relevant evidence.

Year	First Author	Journal	*n* (Type)	Key Remarks	Reference
2021	Hines	Surg Neurol Int	2 (DAW, scalpel sign)	Rapid onset, syrinx, resection	[9]
2021	Rodrigues	Surg Neurol Int	2 (DAW, scalpel sign)	Thoracic DAW, resection	[18]
2022	Delgardo	Neurosurgery	17 (DAW, scalpel sign)	17 pts, 3D-CISS, surgery	[19]
2022	Voglis	Spine J	12 (DAW, scalpel sign)	Multicenter, favorable outcomes	[20]
2022	Ruella	World Neurosurg	7 (scalpel sign)	Mixed DAW/cyst/herniation	[21]
2023	Weng	Int J Neurosci	1 (DAW, scalpel sign)	Occult DAW, 5y follow-up	[22]
2024	Naggar	Egypt J Radiol Nucl Med	197 (DAW, scalpel sign predominantly observed)	Systematic review PRISMA Scalpel sign = highly specific imaging marker for DAW, not exclusive; early detection with 3D-CISS improves diagnosis, CSF flow assessment, and surgical planning.	[17]
2025	Na	Brain Spine	17 (DAW, scalpel sign)	Cord compression, syrinx, surgery	[23]
2025	Asghar	Neurology	1 (DAW, scalpel sign)	DAW case, scalpel sign, resection	[24]
2025	Zahoor	Neurology	1 (scalpel sign)	Arachnoid cyst with compression	[25]
2025	Capo	Neurochirurgie	1 (scalpel sign)	MRI vs surgery correlation	[26]

## Data Availability

The original data presented in this study are available on reasonable request from the corresponding author. The data are not publicly available due to privacy concerns.

## Abbreviations

The following abbreviations are used in this manuscript:

3D-CISS	Three-Dimensional Constructive Interference in Steady State
DAW	Dorsal Arachnoid Web
CSF	Cerebrospinal Fluid
MRI	Magnetic Resonance Imaging
CT	Computed Tomography
